# The Role of Peroxisome Proliferator-Activated Receptors in the Development and Physiology of Gametes and Preimplantation Embryos

**DOI:** 10.1155/2008/732303

**Published:** 2008-02-28

**Authors:** Jaou-Chen Huang

**Affiliations:** Division of Reproductive Endocrinology and Infertility, Department of Obstetrics, Gynecology and Reproductive Sciences, University of Texas Medical School at Houston, 6431 Fannin Street, Houston, TX 77030, USA

## Abstract

In several species, a family of nuclear receptors, the peroxisome proliferator-activated receptors (PPARs) composed of three isotypes, is expressed in somatic cells and germ cells of the ovary as well as the testis. Invalidation of these receptors in mice or stimulation of these receptors in vivo or in vitro showed that each receptor has physiological roles in the gamete maturation or the embryo development. In addition, synthetic PPAR *γ* ligands are recently used to induce ovulation in women with polycystic ovary disease. These results reveal the positive actions of PPAR in reproduction. On the other hand, xenobiotics molecules (in herbicides, plasticizers, or components of personal care products), capable of activating PPAR, may disrupt normal PPAR functions in the ovary or the testis and have consequences on the quality of the gametes and the embryos. Despite the recent data obtained on the biological actions of PPARs in reproduction, relatively little is known about PPARs in gametes and embryos. This review summarizes the current knowledge on the expression and the function of PPARs as well as their partners, retinoid X receptors (RXRs), in germ cells and preimplantation embryos. The effects of natural and synthetic PPAR ligands will also be discussed from the perspectives of reproductive toxicology and assisted reproductive technology.

## 1. INTRODUCTION

Peroxisomes are organelles in eukaryotes that remove toxic substances and break down fatty acid. Peroxisome proliferator-activated receptor *α* (PPAR*α*) was discovered during the search for a compound that increases the proliferation of peroxisomes in mouse liver cells [1]. Subsequently, two additional isotypes, PPAR*β* (also known as PPAR *δ*) and PPAR*γ*, were discovered. The three PPARs are encoded by different genes. Variants arising from alternative splicing and usage of different promoters have been reported in all three PPARs [2]. Together they form a subfamily within the steroid receptor superfamily. To date, PPARs have been identified in many species, including *Xenopus*, sea squirt, zebrafish, *Aedes aegypti* (yellow fever mosquito), *Anopheles gambiae* (a species complex which contains six vectors of malaria), mouse, rat, hamster, and human (http://www.ensembl.org/index.html).

Since their discovery, a great deal has been learned about PPAR*α*, PPAR*γ*, and, to a less extent, PPAR*β*/*δ*. The knowledge has been applied to clinical practice: synthetic PPAR*α* ligands (fibrates) and PPAR*γ* ligands (thiazolidinediones TZD), respectively, are widely used to treat lipid and glucose disorders. In contrast, the use of PPARs to enhance fertility is constrained by our relative meager knowledge regarding PPARs and reproduction. PPAR*γ* activators have recently been used to induce ovulation in women with polycystic ovary disease, a condition of ovulation dysfunction associated with insulin resistance. This review will focus on the roles of PPARs in the development and physiology of gametes and preimplantation embryos. Also included in the discussion are potential impacts of natural or synthetic PPAR ligand on reproduction and the promising benefits of synthetic PPAR ligands in enhancing the success of assisted reproductive technology.

## 2. PPARs AND RXRs

PPARs, similar to steroid and thyroid hormone receptors, are ligand-activated nuclear transcription factors. Unlike steroid and thyroid hormone receptors, PPARs were discovered before their functions were fully understood. Over the years, tissue distribution and synthetic ligands, which bind to specific PPAR, helped to elucidate the biological functions of PPARs.

PPARs form heterodimers with another nuclear receptor, retinoid X receptor (RXR). This interaction occurs in the presence and absence of PPAR ligand. The PPAR-RXR complex recruits other cofactors before binding to PPAR responsive element (PPRE) at the promoter regions of PPAR-responsive genes. Besides PPARs, RXR also forms heterodimers with other nuclear receptors. RXR has three isotypes: RXR*α*, RXR*β*, and RXR*γ*, all of which are activated by 9-*cis*-retinoic acid (but not by all-*trans* retinoic acid) [3]. The 9-*cis*-retinoic acid was originally considered as the endogenous ligand for RXRs in vivo; but recent reports [4, 5] cast considerable doubt that it is the case. Although RXRs exist as three isotypes, they do not confer different functions to PPAR-RXR complexes. The PPAR-RXR complexes are activated by either PPAR or RXR ligand, but simultaneous binding by both ligands elicits more potent activities [6]. A unique feature of PPAR*β*/*δ*, not seen in PPAR*α* or PPAR*γ*, is its ability to repress the transcriptional activities of PPAR*α* and PPAR*γ*. This activity is mediated by corepressors recruited by PPAR*β*/*δ* [7].

The DNA sequence of PPRE is typically of a direct repeat 1 (DR1) nuclear receptor in that the PPRE DNA sequence consists of two repeats of AGGTCA separated by one nucleotide (AGGTCA N AGGTCA). Detailed analyses of native PPREs show that the consensus PPRE sequence is 5^′^-AACTAGGNCA A AGGTCA- 3^′^ [6]. The extended 5^′^ half site, the one imperfect DR1 core, and the adenine as the spacing nucleotide may confer additional selectivity to the binding of PPAR-RXR complex.

## 3. DISTRIBUTION AND BIOLOGICAL FUNCTIONS OF PPAR

The functions of PPARs can be extrapolated from tissue(s) expressing the specific PPAR isotype or from the functions of genes regulated by specific PPAR. PPAR*α* is expressed most abundantly in brown adipose tissue and liver, followed by the kidney, heart, and skeletal muscle. PPAR*γ* is mainly expressed in adipose tissue and, to a less extent, in the colon, the immune system, and the retina. Both PPAR*α* and PPAR*γ* responsive genes are involved in lipid homeostasis. Therefore, it is not surprising that the main functions of PPAR*α* and PPAR*γ* are in glucose and lipid homeostasis [6, 8].

On the other hand, the ubiquitous distribution of PPAR*β*/*δ* (although gut, kidney, and heart express higher levels than other tissues) makes it difficult to associate PPAR*β*/*δ* with specific biological function [8]. The multiple functions of PPAR*β*/*δ* are revealed by the diverse genes regulated by PPAR*β*/*δ*, such as ILK [9], 11 ß hydroxysteroid dehydrogenase II [10], PTEN [9], and 14-3-3*ε* [11]. It is worth noting that 14-3-3*ε* functions as a protein chaperone. Therefore, PPAR*β*/*δ* is indirectly associated with even more diverse range of functions. Indeed, PPAR*β*/*δ* has been implicated in embryo implantation [12], intestinal adenoma [13], colon cancer [14], skin wound healing [15], hair follicle development [16], and cytoprotection [11].

## 4. PPAR LIGANDS

Natural and synthetic PPAR ligands relevant to this review are listed below. More extensive lists are available in the literature [6, 17].

Unsaturated fatty acids are ligands to all PPARs, with PPAR*α* exhibiting the highest affinity; saturated fatty acids, on the other hand, are not effective PPAR ligands. Eicosanoids derived from arachidonic acid form a unique group of fatty acids that bind to PPARs. They include leukotrienes, hydroxyeicosatetraenoic acids (HETEs) (both are formed via the lipoxygenase pathway), and prostaglandins (PGs) (formed via the cyclooxygenase pathway). Leukotriene B4 and 8(S)-HETE are PPAR*α* ligand; and 15-deoxy-Δ12, 14-PGJ_2_ (15d-PGJ_2_, a PGD_2_ derivative) is a PPAR*γ* ligand. Synthetic PPAR*α* (fibrates) and PPAR*γ* (TZD) ligands are used to lower blood lipid and glucose, respectively. Prostacyclin (PGI_2_) is a natural PPAR*β*/*δ* ligand, indeed the uterine PGI_2_ generated by cyclooxygenase-2 (COX-2) mediates the implantation of embryos via PPAR*β*/*δ* [12]. Synthetic PGI_2_ analogs,such as iloprost and carbaprostacyclin, may function as PGI_2_ receptor agonists or PPAR *β*/*δ* ligands. Although iloprost is used as a PGI_2_ receptor agonist to treat pulmonary hypertension and peripheral vasculardiseases, no PGI_2_ analog has been used as a PPAR*β*/*δ* ligand clinically. A recent report indicates that retinoic acid, in cells with high fatty acid binding protein 5 to retinoic acid binding protein-II ratio, may function as a natural PPAR*β*/*δ* ligand [18]. This finding may have evolutional or developmental significance in germ cell maturation, gamete function, or embryo development. PGI_2_ and retinoic acid may provide functional redundancy to ensure PPAR*β*/*δ* activation or they may compliment each other to activate PPAR*β*/*δ* in a developmental stage-dependent manner based on the ratio of the two binding proteins.

## 5. PPAR LIGANDS IN THE REPRODUCTIVE TRACT

Zygotes remain in the oviduct after fertilization and develop to morula or early blastocyst stage embryos before entering into the uterus. It is generally accepted that, compared with cultured embryos (derived from fertilized eggs in vitro or flushed from oviducts at earlier developmental stage), in vivo embryos develop better and have less cell death because oviducts protect the embryos and promote their development [19]. The unique environment provided by the oviduct includes oviduct-derived soluble factors and embryo-derived autocrine factors. Both oviducts and embryos are sources of PPAR ligand(s).

Earlier studies show that the oviduct produces abundant PGE_2_ and PGF_2_ *α*, which regulate its motility. We serendipitously discovered that human [20] and mouse [21] oviducts produce other eicosanoids that activate PPARs. PGI_2_ (a PPAR*β*/*δ* ligand) is the most abundant product, PGD_2_ (whose derivative, 15d-PGJ_2_, is a PPAR*γ* ligand), and other products derived from the lipoxygenase pathway are also produced in substantial amounts. PGI_2_ synthesis by mouse oviducts is synchronized with estrus cycles (and, thus, the development of preimplantation embryos). Peak PGI_2_ synthetic capacity coincides with the window of receptivity, that is, between the eight-cell and morula stages [21, 22].

Recent reports indicate that human [23] and mouse [24, 25] preimplantation embryos express COX isoenzymes and synthesize eicosanoids. PGI_2_ is the most abundant metabolite when radio-labeled arachidonic acid is incubated with blastocyst-stage mouse embryos. Other eicosanoids, such as HETEs and PGD_2_ are also produced by mouse blastocysts [24].

## 6. RXR IN GAMETES AND PREIMPLANTATION EMBRYOS

Gametes and preimplantation embryos express RXRs. Whereas RXR*γ*-null mice are normal [26], RXR*α*- [27] and RXR*β*-null mice [28] have distinctive phenotypes. Gene knockout studies show that spermatogenesis requires RXR*β*. Similarly, oocyte development may be modulated by RXR, which is expressed in both granulosa-cumulus cells and oocytes. Finally, the quality of embryo development may be associated with RXR expression.

### 6.1. RXR in gametes

RXR*α* and RXR*β* are expressed in human cumulus granulosa cells [29] and bovine oocytes [30]. Although the initial reports on RXR*α* [27] and RXR*β* [28] null mice did not include a description of female reproduction (such as follicular development and ovulation), the localization of RXR*α* and *β* in the ovary supports their roles in follicular maturation and oocyte function. RXR may regulate oocyte development directly (via modulating steroidogenesis in the granulosa cells) or indirectly (by affecting oocyte gene transcription) [31]. It is likely that female mice with targeted RXR deletion may suffer subfertility.

The male sterility observed in RXR*β*-null mice [28] underscores the essential role of RXR (and its functional partner) in spermatogenesis. In mouse testes, retinoic acid receptors (RARs) and RXR are expressed in well-defined cell populations: RAR*α* and RXR*β* in Sertoli cells, RAR*β*, RXR*α*, and RXR *γ* in steps 7 and 8 spermatids, and RAR*γ* in spermatogonia. Mouse spermatocytes, however, do not express RARs [32]. Although RAR*β*, RXR*α*, and RXR*γ* are coexpressed in step 7 and 8 spermatids, RAR*β* may not functionally couple with either RXR*α* or RXR*γ*, because RAR*β*-, RXR*γ*-, and RAR*β*/RXR*γ*-null mice do not display reproductive defects [32]. On the other hand, RXR*β* and RAR*α* may form heterodimer and control spermiation in vivo because both are coexpressed in Sertoli cells and invalidation of either gene in mice leads to similar phenotype [32]. RXR*β*-null males are sterile due to oligoasthenoteratozoospermia caused by failed spermatid release (from the germinal epithelium) and abnormal sperm acrosomes and tails [27]. In Sertoli cells, the function of RXR*β* (coupled with RAR*α*) may involve lipid metabolism or transport, because they progressively accumulate lipids (which are unsaturated triglycerides) in RXR*β*-null mice. In older RXR*β*-null males, germ cells degenerate completely and seminiferous tubules are filled with lipid vacuoles [27]. RAR*α* homozygous mutant [33] and mice with targeted RAR*α* ablation in Sertoli cells [34] display similar phenotype. Both have testicular degeneration, failed spermiation, epithelial vacuolation, germ cell desquamation, and apoptosis [34]. Although there is no report concerning RXR expression in spermatozoa, it can be inferred that human sperm express RXR because human sperm express PPAR*γ* (which forms functional complex with RXR) and PPAR*γ* ligand enhances their activities [35].

### 6.2. RXR in preimplantation embryos

The development of preimplantation embryos was not described in the initial reports describing RXR*α*- [27] and RXR*β*- [28] null mice. However, available information in the literature shows that preimplantation embryos express RXRs. Transcripts of RXR*α*, RXR*β*, and RXR*γ* are expressed in zebrafish embryos at 1.5 hour postfertilization [36]. RXR*α*, RXR*β*, and RALDH_2_ (one of the two enzymes oxidizing retinol to retinoic acid) are detected in all stages of preimplantation bovine embryos, including blastocysts which express RXR*β* protein in the inner cell mass and the trophectoderm [30]. RXR*α*, *β*, and *γ* transcripts in preimplantation bovine embryos are likely of maternal origin because eight-cell stage and earlier embryos have significantly higher RXR levels than later stage embryos [37]. Furthermore, RXRs may be essential for optimal embryo development because “good-quality” embryos express significantly higher levels of RXR transcripts than “bad-quality” embryos [37]. It can be summarized that RXR expression in preimplantation embryos described above is corroborated by the expression of its partner, PPAR (discussed later). Furthermore, RXR (partners with PPAR or RAR) is crucial to normal embryo development because (1) early stage embryos contain high levels of maternal RXR mRNA, and (2) “good-quality” embryos express higher RXR mRNA levels.

## 7. PPAR IN GAMETES AND PREIMPLANTATION EMBRYOS

Compared with their role in postimplantation embryo development, the roles of PPARs in fertilization, implantation, and embryo development are less well defined. Available information does suggest that gametes and preimplantation embryos express functional PPARs and that PPAR activation optimizes their functions.

### 7.1. PPAR and gametes

All three PPAR isotypes are expressed in somatic and germ cells of the testis. In rat, PPAR*α* and *β*/*δ* are expressed in Leydig cells and Sertoli cells [38]. In human, PPAR*γ*1 message is detected in the testis [39]. In mouse, both PPAR*α* and *γ* are expressed in Sertoli cells [40], and PPAR*β*/*δ* is expressed in spermatids and spermatocytes [41]. The expression of PPAR*β*/*δ* in mouse spermatids and spermatocytes is further supported by the expression of *Ssm*, a novel PPAR*β*/*δ* target gene, in mouse testis [42]. The functionality of PPAR*α* in Sertoli cells is confirmed by its nuclear translocation in response to a selective PPAR*α* ligand, Wy-14,643 [40]. These findings suggest that PPARs (in Sertoli cells and Leydig cells) provide an environment for spermatogenesis and may be directly involved in germ cell maturation. PPAR may regulate germ cell maturation in a stage-dependent fashion. In zebrafish, PPAR*γ* is expressed in spermatogonia but not in spermatocytes [43].

PPAR ligand affects spermatogenesis and sperm activities. Di(*n*-butyl) phthalate, a PPAR activator, modulates the expression of genes related to spermatogenesis and steroidogenesis and causes testicular atrophy in rats [44]. In contrast, the capacitation, acrosome reaction, and motility of ejaculated human sperm are enhanced by a treatment with rosiglitazone (a synthetic PPAR*γ* ligand) or 15d-PGJ2 (a natural PPAR*γ* ligand) [35]. Since germ cells express all three PPAR isotypes, the expression and function of two other PPAR isotypes, PPAR*α* and *β*/*δ*, in mature spermatozoa warrant further investigation.

In several species including rat, all three PPAR isotypes are detected in the ovary [2]. PPAR*γ*, which has been studied more extensively than the other two isotypes, is detected in the mouse, rat, pig, sheep, cow, and human ovary. PPAR*γ* is expressed strongly in the granulosa cells of rat [2], mouse [41], and sheep [45], as well as in oocytes from cattle [30], zebrafish [43], *Xenopus* [46], and human [47]. PPAR*γ* is detected in different classes of follicles (primary/secondary to preovulatory follicles) and its expression increases with the development of follicles. After the LH surge, PPAR*γ* mRNA expression is downregulated [2]. Activation of PPAR*γ* by natural and synthetic ligands in the granulosa cells appears to regulate the synthesis of steroid hormones. Thus, PPAR*γ* may be indirectly involved in oocyte maturation via the granulosa cells. Indeed, disruption of PPAR*γ* gene in the ovary using *cre/loxP* technology led to female subfertility [48]. On the other hand, PPARs may be directly involved in oocyte maturation. Indeed, it has been reported that rosiglitazone, a synthetic PPAR*γ* ligand, at 100 *μ*M stimulates AMP-activated protein kinase (AMPK) and enhances the meiotic resumption of mouse oocytes [42].

### 7.2. PPAR and preimplantation embryos

Preimplantation bovine and mouse embryos express PPAR*γ* and PPAR*β*/*δ*, respectively. Beginning at two-cell stage and throughout the preimplantation period, bovine embryos express PPAR*γ*. Blastocyst stage bovine embryos express PPAR*γ* in the inner cell mass and the trophectoderm [30]. Mouse embryos express PPAR*β*/*δ* detectable by immunohistochemistry at two-cell stage [25] or eight-cell stage [22] and throughout the preimplantation period. Mouse blastocysts also express PPAR*β*/*δ* in the inner cell mass and the trophectoderm [22].

Although preimplantation embryo development and implantation were not specifically examined in the initial report regarding PPAR*β*/*δ*-null mouse, the report provides a hint of the impacts of PPAR*β*/*δ* deficiency [49]. The genotypic distribution of embryos on gestation day 9.5 shows that PPAR*β*/*δ*—/— embryos are underrepresented: PPAR*β*/*δ*—/— embryos represent 16% (3/19) and 38% (3/8) of embryos from PPAR*β*/*δ*+ / — x PPAR*β*/*δ*— / — and PPAR*β*/*δ*— / — x PPAR*β*/*δ*+ / — mating, respectively. This represents a 36% (i.e., 25% versus 16%) and a 24% (i.e., 50% versus 38%) deviation from the expected Mendelian frequency. Loss of PPAR*β*/*δ*— / — embryos prior to gestation day 9.5 may occur at any stage including ovulation, fertilization, preimplantation period, implantation, and postimplantation period up to gestation day 9.5. The results of our study show that PPAR*β*/*δ* ablation adversely affects preimplantation embryo development and, consequently, implantation [22]. Compared with wild-type embryos, PPAR*β*/*δ*— / — embryos show developmental delay as early as 48 hours after two-cell stage embryos are harvested. The gap widens in the subsequent 48 hours. At 96 hours after the harvest of two-cell embryos, 100% of wild-type embryos have reached or passed the blastocyst stage (versus 65% PPAR*β*/*δ*— / — embryos), and 85% of wild-type embryos have undergone hatching or hatched completely (versus 28% PPAR*β*/*δ*— / — embryos). Consequently, PPAR*β*/*δ*— / — embryos implant less effectively than wild-type embryos (28% versus 44%). We also found that PPAR*β*/*δ*— / — embryos have decreased embryonic cell proliferation compared with that observed in wild-type embryos. These results suggest that PPAR*β*/*δ* activation via endogenous PPAR*β*/*δ* ligand, such as PGI_2_ [24] and/or retinoic acid [18], confers the “basal” momentum (including cell proliferation and possibly other functions) to preimplantation embryos and propels them through various stages of development.

In addition to providing a “basal” momentum of embryo development via endogenous PPAR*β*/*δ* ligand, PPAR*β*/*δ* activation by synthetic ligand further enhances the development and the implantation of cultured embryos. Both L-165041 (a synthetic PPAR*β*/*δ* ligand) and iloprost (a stable PGI_2_ analog) enhance complete embryo hatching in a concentration-dependent manner [22, 50, 51]. Embryos preconditioned with L-165041 or iloprost show higher implantation rates when transferred to gestational carriers [22, 52]. These results suggest that cultured embryos do not reach their full developmental potential due to insufficient endogenous PPAR*β*/*δ* ligands or lack of exogenous PPAR*β*/*δ* ligands normally provided by the oviduct. Embryos exposed to PPAR*β*/*δ* ligand have increased embryonic cell proliferation compared with controlled embryos [22].

## 8. PPAR AND REPRODUCTIVE TOXICOLOGY

PPAR activators are found in herbicides, industrial plasticizers (for a brief review see [53]), and personal care products such as hair spray and solvent for perfumes [54]. Di(*n*-butyl) phthalate, a PPAR*γ* activator found in plasticizers and personal care products, may cause male infertility by altering hormones involved in steroidogenesis and spermatogenesis [44]. Other potential PPAR activators posing reproductive toxicology concerns are pharmaceutical agents used to lower lipids and blood glucose. Rosiglitazone (a TZD for diabetes) may activate PPAR*γ* ligand and enhance sperm activities in men [35] or, depending on its concentration, may enhance meiosis resumption of oocytes or induce oocyte degeneration in women [55]. Nonsteroidal anti-inflammatory drugs, such as Motrin, which blocks PG synthesis, pose reproductive hazards through a different mechanism. Decreased PG (such as PGI_2_) production may adversely affect embryo development and implantation.

On the other hand, PPARs may be exploited to enhance the success of assisted reproductive technology. The fertilization potentials of human sperm in in vitro fertilization (IVF) or other assisted reproductive technologies, such as artificial insemination, may be enhanced by incubating sperm with synthetic PPAR*γ* ligands. The development and implantation of IVF embryos may be augmented by supplementing culture media with PGI_2_ analogs, synthetic PPAR*β*/*δ* ligand, or retinoic acid. However, potential long-term adverse effects are unknown. Large-scale clinical trials of sufficient power are needed to validate the benefits and to assess the harms.

## 9. CONCLUSION

The literature on PPARs in gametes and preimplantation embryos is relatively limited. Nonetheless, the consensus is that PPAR serves to optimize gamete function and embryo development. Further studies are needed to shed more light on the physiological roles of PPARs in reproduction. The knowledge gained will help us avoid potential reproductive hazards and augment the success of assisted reproductive technologies.
